# MicroRNA-145 engineered bone marrow-derived mesenchymal stem cells alleviated erectile dysfunction in aged rats

**DOI:** 10.1186/s13287-019-1509-1

**Published:** 2019-12-18

**Authors:** Qiwei Liu, Yubin Cui, Haojian Lin, Daoyuan Hu, Tao Qi, Bo Wang, Zhansen Huang, Jun Chen, Ke Li, Hengjun Xiao

**Affiliations:** 1 Department of Urology, The Third Affiliated Hospital of Sun Yat-Sen University, Tianhe Road 600#, Guangzhou, 510630 China; 2 Department of Infertility and Sexual Medicine, The Third Affiliated Hospital of Sun Yat-Sen University, Tianhe Road 600#, Guangzhou, 510630 China

**Keywords:** miR-145, Bone marrow-derived mesenchymal stem cells, Age-associated erectile dysfunction

## Abstract

**Background:**

Aging is one of the dominant factors contributing to erectile dysfunction (ED), and effective treatments for age-associated ED are urgently demanded. In this study, the therapeutic efficiency of bone marrow-derived mesenchymal stem cells (BMSCs) overexpressing microRNA-145 (miR-145) was evaluated in ED.

**Methods:**

Sixty male Sprague-Dawley rats (24 months old) were randomly divided into 4 treatment groups (*n* = 15/group): PBS (control), BMSCs, BMSCs transfected with a blank vector (vector-BMSCs), and BMSCs transfected with a lentivirus overexpressing miR-145 (OE-miR-145-BMSCs). Fourteen days after transplantation of BMSCs, erectile function was evaluated by measuring intra-cavernous pressure (ICP) and mean arterial pressure (MAP). Subsequently, penile erectile tissues were harvested and subjected to Masson staining, qRT-PCR, immunofluorescence staining, dual luciferase assay, and Western blot analysis.

**Results:**

Fourteen days after transplantation, the ICP/MAP was 0.79 ± 0.05 in the OE-miR-145-BMSC group, 0.61 ± 0.06 in the BMSC group, 0.57 ± 0.06 in the vector-BMSC group, and 0.3 ± 0.01 in the PBS group. Treatment with OE-miR-145-BMSCs significantly improved ED (*P* < 0.05), and the treatment increased the smooth muscle content in the penis tissues of ED rats (*P* < 0.05). In the OE-miR-145-BMSC group, the expression levels of α-SMA, desmin, and SM-MHC were higher than they were in the other ED groups (*P* < 0.05). In addition, the levels of collagen 1, MMP2, and p-Smad2 in the BMSC-treated group, especially in the OE-miR-145-BMSC group, were lower than those in the control group (*P* < 0.05).

**Conclusions:**

MicroRNA-145 engineered BMSCs effectively attenuate age-related ED. Transplantation of miR-145-overexpressing BMSCs may provide a promising novel avenue for age-associated ED therapy.

## Background

Erectile dysfunction (ED) is a widespread affliction in aging men, leading to an inevitable decrease in the quality of life [1]. As men age, the prevalence of ED increases [2–4]. However, the underlying mechanisms remain poorly understood. Previous studies suggest that ED in aged men may be associated with decreased smooth muscle content and increased collagen deposition [5]. Phosphodiesterase type 5 (PDE5) inhibitor medications, such as sildenafil and tadalafil, have been broadly applied for the treatment of ED in a wide spectrum of aetiologies and ages [6–10]. However, their application was constrained by some disappointing adverse effects, such as flushing, dyspepsia, and headaches [7]. They are also unavailable to patients with cardiovascular diseases, who frequently use nitrates. A Swedish study has reported that treatment with PDE5 inhibitors shows a mild positive relationship with the occurrence of malignant melanoma in men. PDE5 inhibitor use was also associated with an increased risk of basal cell carcinoma [11]. Therefore, safer and more effective approaches for ED are urgently needed.

Increasing evidence shows that mesenchymal stem cells (MSCs) have self-renewal capacity and the potential to differentiate into a wide range of cell populations in certain settings. MSC-based treatments have been shown to generate positive therapeutic effects in age-related ED [12–14]. MSCs can exert regenerative, anti-apoptosis, and anti-fibrosis effects [15–18]. However, treating ED with MSCs is still in the trial phase, and the detailed mechanisms of MSC action remain elusive.

MicroRNAs have been found to regulate stem cell self-renewal and pluripotency. Cordes et al. [19] have reported a crucial role for miR-145 during the differentiation of stem cells into smooth muscle cells (SMCs). Lack of miR-145 impairs SMC differentiation [20, 21]. Elevated miR-145 levels rescued the expression of SMC markers in SMC-Dicer knockout rats [22].

Herein, BMSCs overexpressing miR-145 were established, and their effects and the mechanisms driving these effects were investigated. We found that BMSCs overexpressing miR-145 increased the number of SMCs by directly targeting Kruppel-like factor 4 (KLF4) and reducing the expression of collagen 1 and matrix metallopeptidase 2 (MMP2); further, the cells reduced the phosphorylation of the mediator of TGF-β signaling, SMAD2 by targeting transforming growth factor beta receptor 2 (TGFBR2). These changes resulted in significantly amplified erectile responses in aged rats. Our data define a positive therapeutic effect from the transplantation of miR-145-overexpressing BMSCs in the treatment of ED.

## Methods

### miR-145-overexpressing BMSCs

Primary rat BMSCs were purchased from Cyagen Biosciences (Guangzhou, China) INC. Cells were maintained in low-glucose medium (DMEM; HyClone, USA) containing 10% fetal bovine serum (FBS; Gibco, USA) and 1% penicillin–streptomycin (Gibco, USA) under standardized conditions (5% CO_2_, 37 °C). Cells were passaged every 2–3 days. Control BMSCs and microRNA-145 engineered BMSCs were used between passages 3 and 4.

Lentiviruses expressing miR-145 (lentivirus-miR-145-GFP) or GFP (lentivirus-vector-GFP) were generated by Hanbio Biotechnology (Shanghai, China). During the transfection, BMSCs were cultured overnight in 6-well plates at a density of 1 × 10^4^/well. Then, BMSCs were trypsinized, centrifuged, and cultured in medium containing lentivirus-miR-145-GFP or lentivirus-vector-GFP at a concentration of 5 × 10^5^/well. After 6 h of transfection, the supernatants were discarded and replaced with fresh cell culture medium. The cells were continuously cultured for 3–5 days in the incubator.

### Cell viability assay

Cells were maintained at 10,000 cells/well in 96-well plates for 24 h at 37 °C and 5% CO_2_. Next, 10 μL of CCK8 solution was mixed into each well, and the cells were incubated for another 2 h. The optical density value was detected at 450 nm by a microplate reader (Biotek Elx08, USA). The experiments were performed in triplicate.

### Animal experiments

Sixty male Sprague-Dawley aged rats (24 months old) selected with apomorphine were randomly divided into 4 treatment groups (*n* = 15/group): PBS (control), BMSCs, BMSCs transfected with blank vector (vector-BMSCs), and BMSCs overexpressing miR-145 (OE-miR-145-BMSCs). Animals were anesthetized by injection with 2% pentobarbital sodium (40 mg/kg; Beyotime, China) into the peritoneal cavity. After ligating the base of the penis with the rubber band, rats in the control group were intra-cavernously treated with 200 μL of PBS solution, rats in the BMSC group were intra-cavernously transplanted with 1 × 10^6^ BMSCs in 200 μL of PBS, rats in the vector-BMSC group were transplanted with 1 × 10^6^ BMSCs infected with the blank vector in 200 μL of PBS, and rats in the OE-miR-145-BMSC group were transplanted with 1 × 10^6^ miR-145-overexpressing BMSCs in 200 μL of PBS. Five minutes after the operation, the rubber band was removed. All experimental protocols were approved by the Committee of Animal Care and Use in Sun Yat-sen University.

### Measurement of erectile function

At 14 days after injection, erectile function was assessed. Animals were anesthetized with 2% pentobarbital sodium (40 mg/kg) and incised along the midline neck and abdomen to expose their carotid arteries and cavernous nerves, respectively. The artery was cannulated, and a tubing lined with heparin was attached to record its mean pressure. Then, the epidermis of the penis was removed, and its crura were identified. A 25-gauge needle was inserted into the corpora cavernosum of the penis. We applied a bipolar electrode to stimulate the nerves, which was connected to a multi-channel signal producer (BL-420F, Taimeng, China). The relevant parameters were 5 V, 25 Hz, pulse width of 5 ms, and duration of 60 s. A signal-obtaining framework was utilized to record the mean arterial pressure (MAP) and intra-cavernous pressure (ICP). After the measurements, the rats were euthanized by injecting air into the common carotid artery, and then, the penile tissues were resected for subsequent analyses.

### Masson staining

The collected penis samples were fixed in 4% paraformaldehyde for 24 h at 4 °C and were then embedded in paraffin blocks. Slices were dewaxed and dyed for 20 min using Weigert’s iron hematoxylin. Sections were then washed with demineralized water and dyed with Biebrich scarlet-acid fuchsin. Subsequently, the sections were dyed with phosphomolybdic-phosphotungstic acid and aniline blue. After treating with 1% acetic acid, the samples were dried and mounted. Relative amounts of smooth muscle and collagen were measured by an image analyzing system.

### Quantitative real-time PCR

Total RNA was extracted using a TRIzol reagent kit (Thermo Fisher Scientific, USA). RNA was reverse transcribed using the PrimeScript® RT reagent Kit (TaKaRa, Tokyo, Japan). Then, qRT-PCR was conducted using a PRISM 7500 System (Applied Biosystems, Waltham, MA, USA) with a SYBR Premix ExTaqII (TliRNaseHPlus) kit (Takara). The experimental data were processed by the 2^−ΔΔCt^ method. The expression of relative genes was normalized to β-actin. All primers are listed in Table 1.

**Table 1:** Primer sequences used for qRT-PCR

Genes	Primer sequences (5′→3′)
miR-145	F: GTTCGTGCGGTCCAGTTTTCCCAG
	R: GTCGTATCCAGTGCAGGGTCCGAG
MMP2	F: GCCCAGAGACTGCTATGTCCA
	R: CCCACTTCCGGTCATCATCGT
CollagenI	F: CTGGTGCCAA-AGGAGAACCC
	R: CCATCAGCACCAGGGAAACC
α-SMA	F: GGCCGAGATCTCACCGACTA
	R: GCAGCAGT-GGCCATCTCATT
Desmin	F: CTTCCGAGCGGATGTGGATG
	R: GGACCTGCTGTTCCTGAAGC
SM-MHC	F: AATG-AGGCAGAAGGAAGGC
	R: TCCTCATCCAGCTGGTCCTG
β-actin	F: CGTGAAAAGATGACCCAGATCA
	R: CAGCCTGGATGGCTACGTACA

### Western blot analysis

The collected penile specimens were lysed in RIPA buffer (Cell Signaling Technology, USA) containing a protease inhibitor cocktail (KeyGEN BioTECH, China). The proteins were separated by SDS-PAGE and transferred to a PVDF membrane (Millipore, USA). After blocking, the membrane was incubated with primary antibodies against desmin (Abcam, USA), SM-MHC (Abcam, USA), Collagen1 (Abcam, USA), MMP2 (Abcam, USA), p-SMAD2 (Abcam, USA), SMAD2 (Abcam, USA), and β-actin (Abcam, USA). After washing the membrane three times, appropriate secondary antibodies were applied and incubated with the membrane for 1 h. Protein bands were detected by ECL chemiluminescence reagents (KeyGEN BioTECH, China) and imaged with an imaging system (UVP GDS-8000, USA).

### Immunohistochemistry and immunofluorescence

Slices were dewaxed and hydrated utilizing EDTA solutions. After blocking with serum for 30 min, the slices were incubated with antibodies, which include anti-α-SMA (Cell Signaling Technology, USA), anti-collagen 1 (Abcam, USA), or anti-MMP2 (Abcam, USA) at 4 °C overnight. After washing with PBS, the slices were incubated with appropriate secondary antibodies for 50 min at 37 °C. After rinsing with PBS, diamidino-phenyl-indole (DAPI) was applied to stain the nuclei. Images were photographed by microscopy (Nikon, Japan).

### Luciferase assays

Wild-type and mutant KLF4 (with mutated miR-145 binding sites) were cloned into a pmirGLO dual luciferase vector (GenePharma) to construct dual luciferase reporter plasmids. HEK293T cells were cotransfected with wild-type pmirGLO-KLF4 (or mutant) and miR-145 mimics (or a negative control) using Lipofectamine 2000. Similarly, dual luciferase reporter plasmids containing TGFBR2-WT and TGFBR2-Mut were constructed. HEK293T cells were cotransfected with either miR-145 mimics/inhibitor or their corresponding empty vectors and luciferase reporter plasmids using Lipofectamine 2000. Luciferase activity was analyzed using a dual luciferase reporter kit (Promega, USA) after 48 h of transfection. Renilla luciferase activity was measured as a transfection efficiency control.

### Statistical analysis

The results are presented as the means ± SD and were analyzed using SPSS version 20. Statistical analysis was performed using one-way ANOVA and Dunnett’s tests. The results were considered to be significant at *P* < 0.05.

## Results

### Establishment of BMSC overexpressing miR-145

**Fig. 1:**
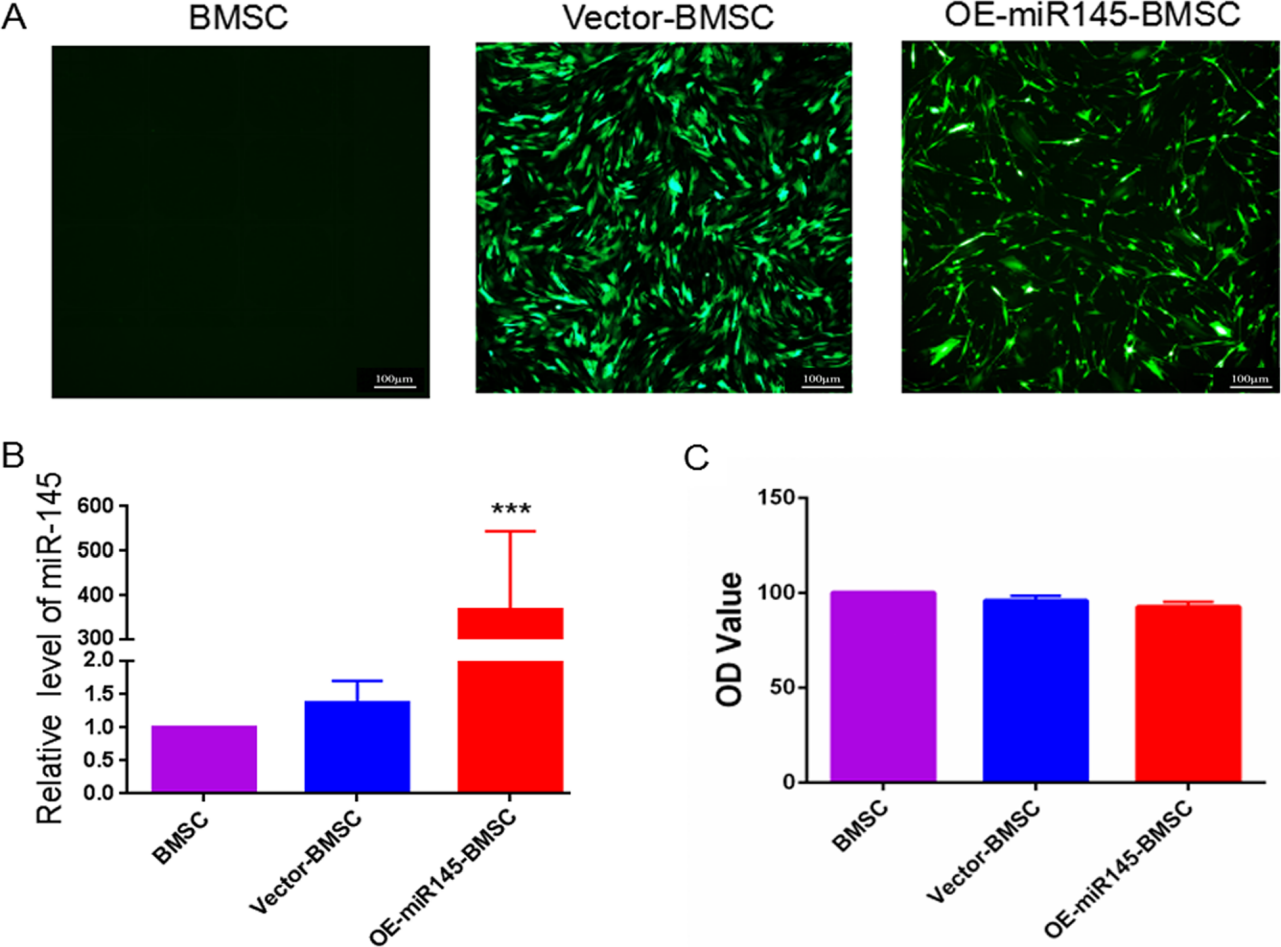
Construction of BMSCs overexpressing miR-145. **a** Forty-eight hours after transfection, control cell, BMSC, vector-BMSC, and OE-miR-145-BMSC groups were detected by fluorescence microscopy. **b** The expression level of miR-145 was measured by qPCR. **c** The viability of BMSCs among the groups was assessed by using CCK8 assays. LV lentivirus; NC negative control. ***P < 0.001 compared with the control group

BMSCs were efficiently infected with a lentivirus expressing miR-145/GFP (Fig. 1a). The transduced ratio was almost the same between cells transduced with LV-miR-145-GFP and those transduced with LV-vector-GFP. qRT-PCR analysis revealed a marked increase in miRNA expression after infection with the miR-145-overexpressing lentivirus (Fig. 1b). A CCK8 assay was used to assess the effect of miR-145 on the viability of BMSCs. The results showed that overexpression of miR-145 had no significant effect on the viability of BMSCs (Fig. 1c).

### miR-145-overexpressing BMSCs effectively augment the erectile response

**Fig. 2:**
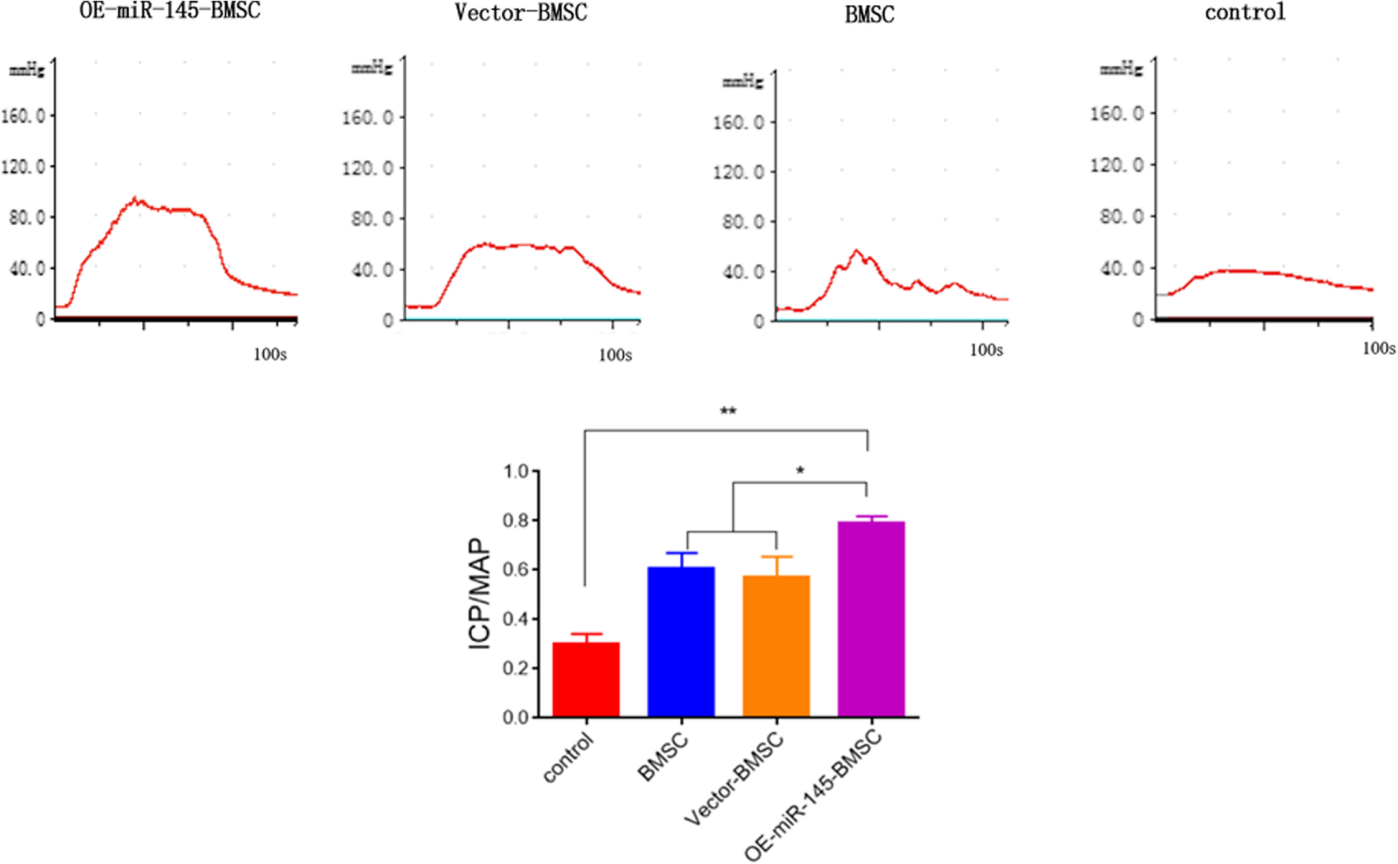
Erectile response at 14 days after injection with BMSCs. Images of ICP and ICP/MAP in the control cell, BMSC, vector-BMSC, and OE-miR-145-BMSC groups. *P < 0.05, **P < 0.01 compared with the control group

Intra-cavernous pressure analysis showed that at 14 days after injection with BMSCs, the ICP/MAP was 0.79 ± 0.05 in the OE-miR-145-BMSC group, 0.61 ± 0.06 in the BMSC group, 0.57 ± 0.06 in the vector-BMSC group, and 0.3 ± 0.01 in the PBS group. The ICP/MAP values were significantly higher in the BMSC-treated groups than those in the control group, while treatment with BMSCs overexpressing miR-145 further increased the value of ICP/MAP (Fig. 2, *P* < 0.05).

### BMSCs overexpressing miR-145 increase smooth muscle content in penis tissues

**Fig. 3:**
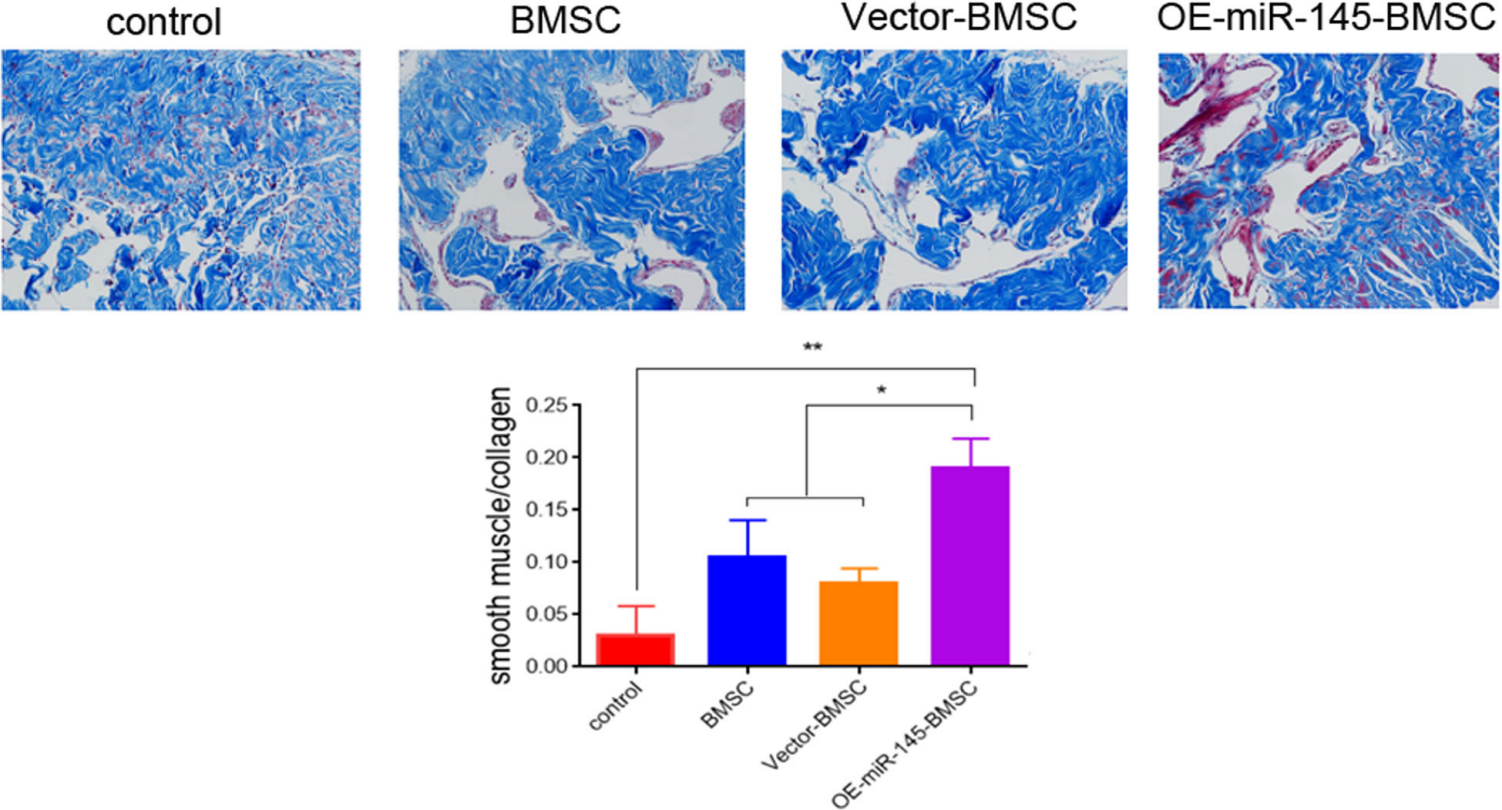
Representative images of Masson staining in penis tissues. The ratio of smooth muscle (red) to collagen (green) among the control cell, BMSC, vector-BMSC, and OE-miR-145-BMSC groups at 14 days after transplantation. *P < 0.05, **P < 0.01 compared with the control group

Relative quantities of smooth muscle and collagen were determined by Masson staining. At 14 days after implantation, the ratios of SMC-to-collagen were 0.21 ± 0.04 in the OE-miR-145-BMSC group, 0.1 ± 0.07 in the BMSC group, 0.08 ± 0.02 in the vector-BMSC group, and 0.03 ± 0.007 in the PBS group (Fig. 3, *P* < 0.05). The OE-miR-145-BMSC group had significantly higher SMC-to-collagen ratio than the other groups, indicating the recovery of smooth muscle content after BMSC treatment.

### BMSCs overexpressing miR-145 increase markers of smooth muscle

**Fig. 4:**
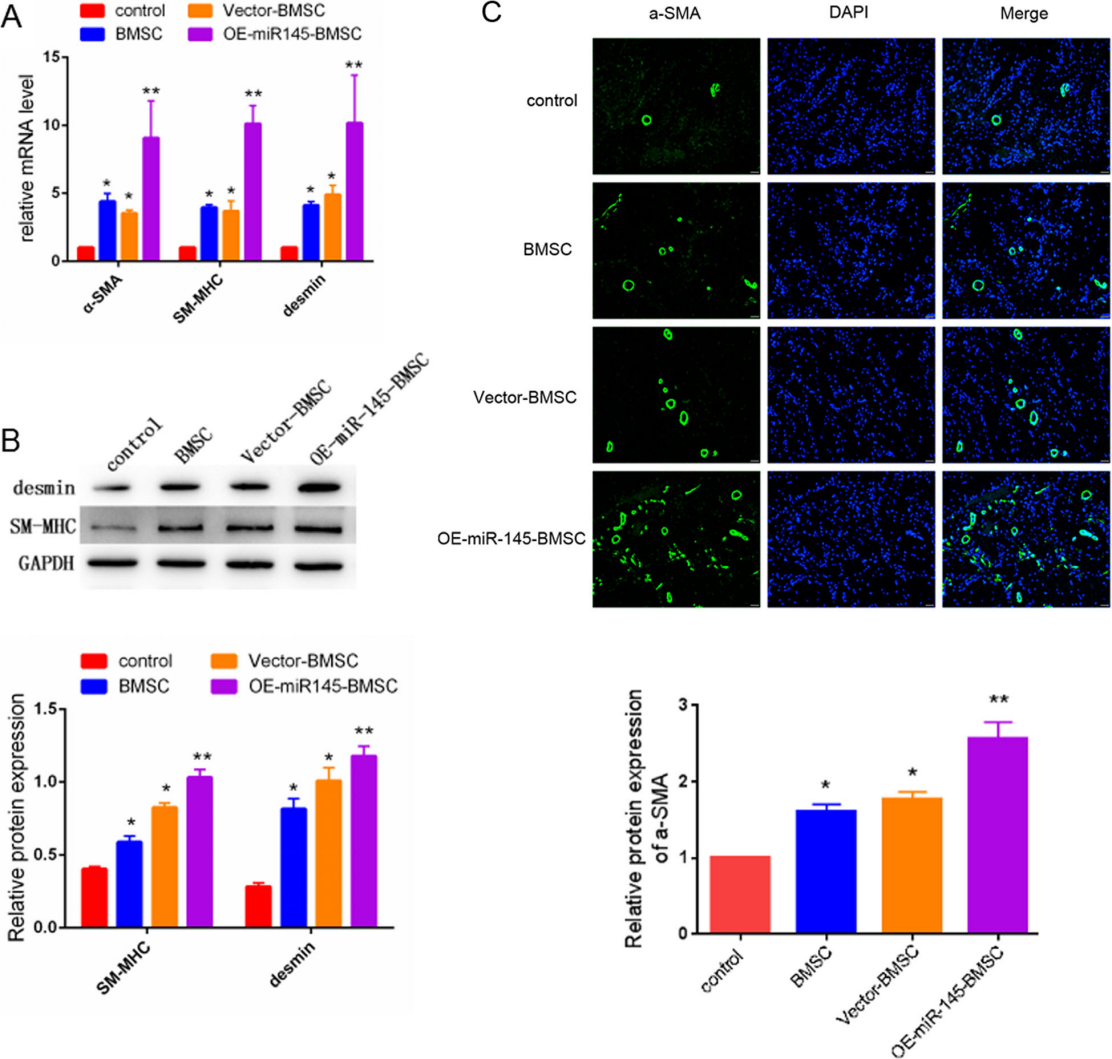
Representative images of smooth muscle cell marker expression at 14 days after injection. **a** mRNA expression levels of α-SMA, desmin, and SM-MHC were measured by qRT-PCR. **b** Protein expression levels of desmin and SM-MHC were detected by Western blot. **c** Immunofluorescence images of α-SMA in penis tissues. Green represents α-SMA. Cell nuclei were stained with DAPI (blue). *P < 0.05, **P < 0.01 compared with the control group

After 14 days of manipulation, penile specimens were immunostained and underwent Western blotting to investigate the expression of SMC markers. As shown in Fig. 4a, the mRNA levels of α-SMA, desmin, and SM-MHC were markedly higher in the OE-miR-145-BMSC group than those in the other groups. Western blotting analysis showed that the protein levels of desmin and SM-MHC in the penis tissue were highest in the OE-miR-145-BMSC group, followed by the BMSC group, the vector-BMSC group, and the control group (Fig. 4b, *P* < 0.05). Consistent with the above findings, immunofluorescence staining showed that α-SMA expression was obviously increased in BMSC-treated groups compared with the control group and that injection with BMSCs overexpressing miR-145 resulted in the highest level of α-SMA.

### BMSCs overexpressing miR-145 decreases collagen 1 and MMP2 expression

**Fig. 5:**
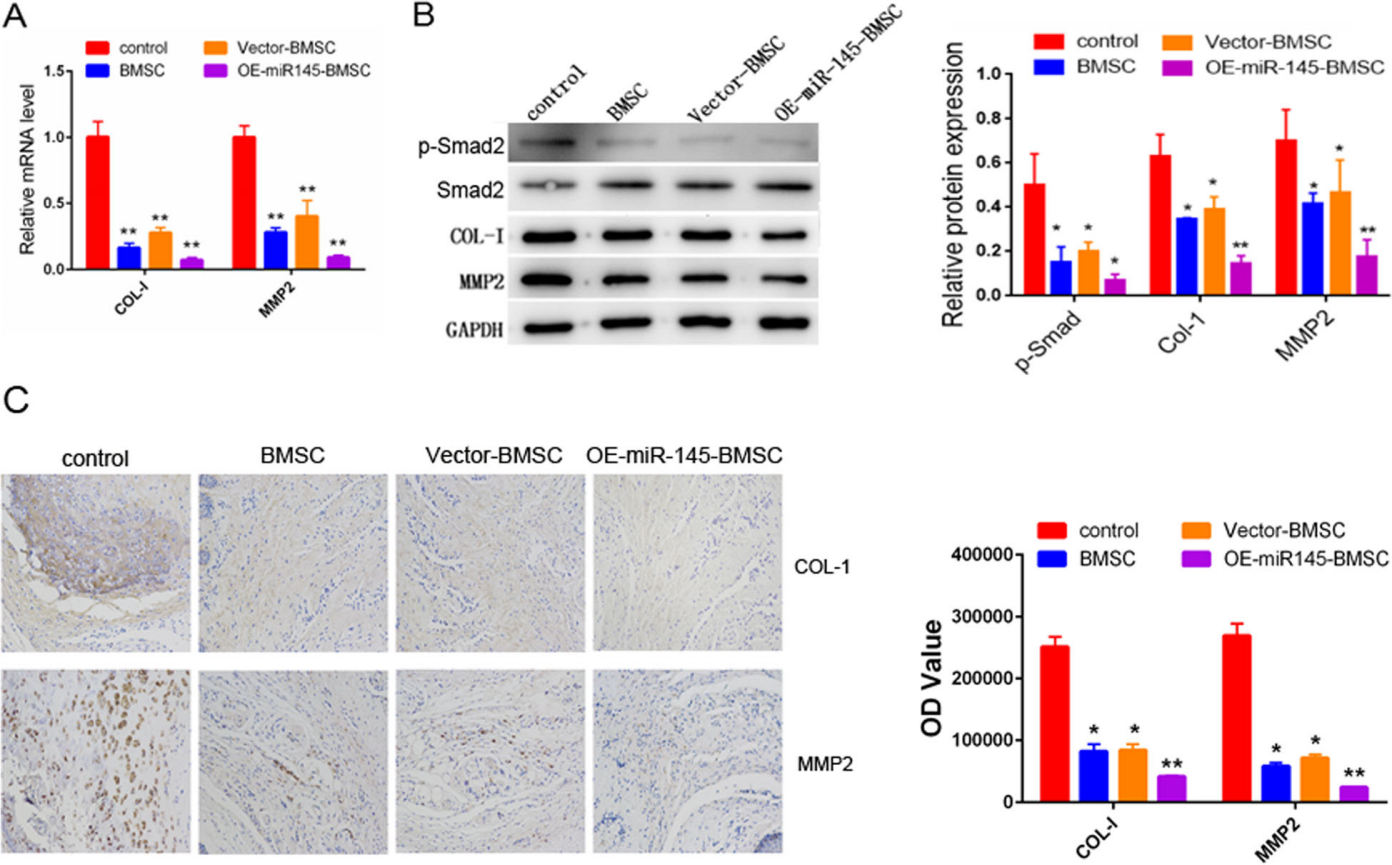
Representative images of collagen 1 and MMP2 expression. **a** qRT-PCR analysis of collagen 1 and MMP2 in penis tissues from each group. **b** Western blot analysis of collagen 1, MMP2 p-SMAD2, and SMAD2 in penis tissues from each group. **c** Immunohistochemistry analysis of collagen 1 and MMP2 in control cell, BMSC, vector-BMSC, and OE-miR-145-BMSC groups are presented. *P < 0.05, **P < 0.01 compared with the control group

The expression levels of collagen 1 and MMP2 at 14 days after injection were determined by real-time PCR, Western blot, and immunohistochemical assays (Fig. 5a–c). The results showed that the mRNA and protein levels of collagen 1 and MMP2 declined markedly in the OE-miR-145-BMSC group compared with the other groups (*P* < 0.05). Moreover, we determined the expression of TGF-β mediators by Western blotting with the specimens from 14 days after injection. As shown in Fig. 5b, compared with the control group, BMSC-treated groups, especially the OE-miR-145-BMSC group, showed obviously decreased phosphorylation of SMAD2 (*P* < 0.05).

### KLF4 and TGFBR2 are downstream targets of miR-145

**Fig. 6:**
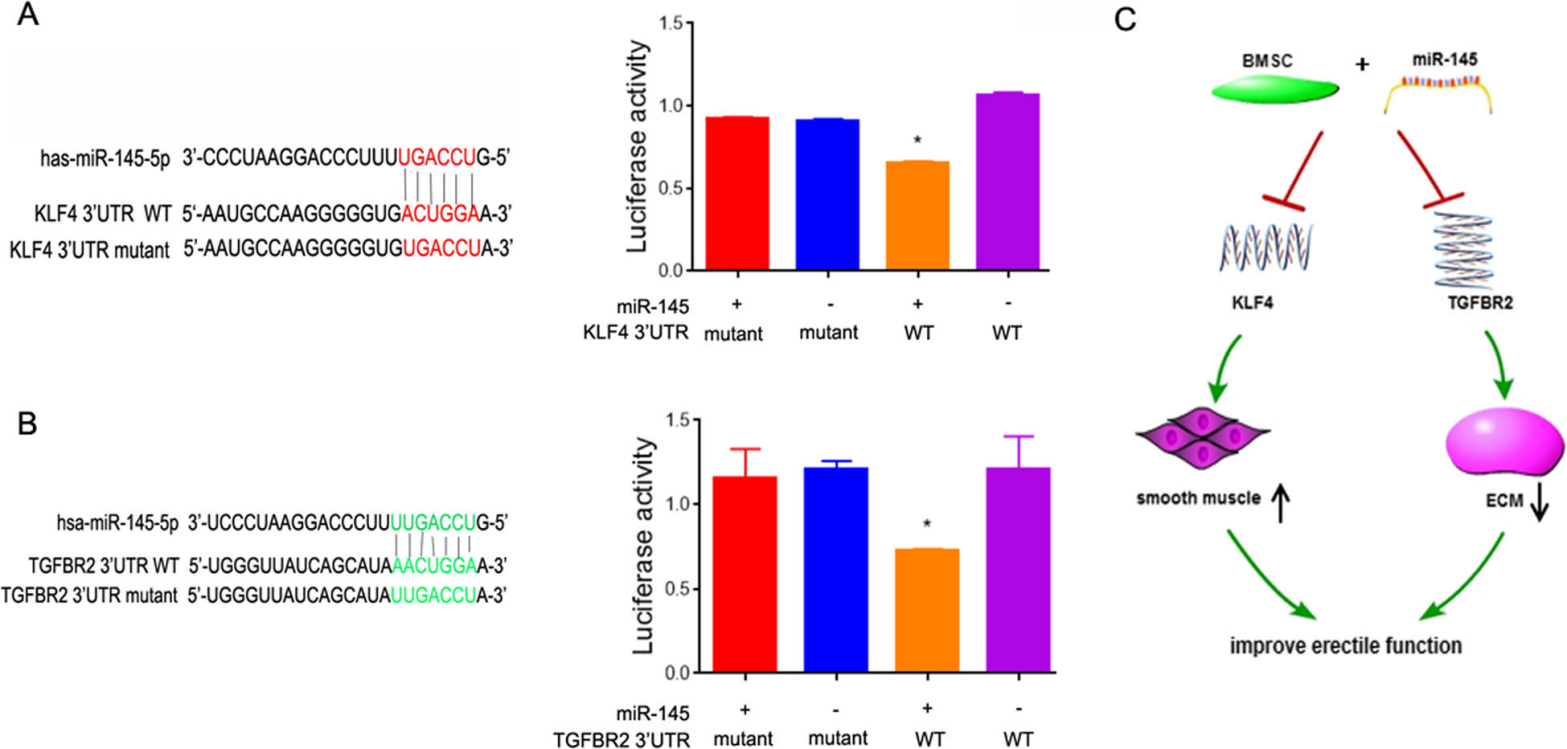
KLF4 and TGFBR2 are targets of miR-145. **a** The predicted binding sites between miR-145 and KLF4 are shown, and luciferase reporter assay results are shown for BMSCs transfected with a reporter containing a KLF4 3′UTR (wild-type/mutant) and a miR-145 (NC/mimic). **b** The predicted binding sites between miR-145 and TGFBR2 are shown, and luciferase reporter assay results are shown for BMSCs transfected with a reporter containing a TGFBR2 3′UTR (wild-type/mutant) and a miR-145 (NC/mimic). *P < 0.05, **P < 0.01. **c** The diagram of a proposed mechanism is shown

miRNAs function by binding to target genes. Targetscan and microrna.org were applied to predict possible targets of miR-145, and KLF4 and TGFBR2 were found to be potential targets (Fig. 6a, b). To further confirm the direct interaction between miR-145 and the two predicted target genes, luciferase reporter assays were carried out. As expected, transfection with miR-145 significantly decreased the luciferase activity from the reporter carrying the wild-type KLF4 3′UTR and the wild-type TGFBR2 3′UTR, but miR-145 did not have an effect on the mutant 3′UTRs (Fig. 6a, b).

## Discussion

Aging is a key contributing factor to ED [23]. Drug therapy is a common treatment approach; however, therapeutic efficacy is unsatisfactory. Thus, more effective strategies for aged-associated erectile failure are required. MSC therapy is a promising approach for ED, of which many studies have been undertaken [24–26]. Additionally, several clinical trials using MSC therapy have been reported [27–30]. Nevertheless, more investigations are still needed before this approach is viable and effective. In this study, we found that treatment with miR-145-expressing BMSCs markedly alleviated ED in aged rats, indicating an important role for miR-145 in ED improvement.

miRNAs are a group of non-coding RNAs that function in various biological processes, including modulating ED [31]. For example, Li et al. reported that treatment with a miR-328 antagomir could improve ED in STZ-induced diabetic rats by regulating cGMP and AGEs [32]. Wen et al. also revealed an association between miR-205 and the pathogenesis of diabetes mellitus-induced erectile dysfunction, which was caused by downregulation of AR expression [33]. BMSCs differentiating into endothelial cells have been shown to restore erectile dysfunction [34]. Therefore, facilitating BMSC differentiation may be a promising strategy for improving ED. A number of studies have shown that miRNAs play crucial roles in controlling BMSC differentiation. For instance, miR-125b and miR-320a have been shown to facilitate the differentiation of MSCs by targeting BMPR1b and HOXA10 [35, 36]. MiR-320 promotes the adipocytic differentiation of MSCs by directly targeting RUNX2. Similarly, miR-154 has a vital role in the differentiation of mesenchymal stem cells into vascular SMCs [37], which are important for erectile function [14].

A sufficient amount of relaxed smooth muscle is necessary for erection of the penis. A deficiency of smooth muscle triggers veno-occlusive dysfunction and aggravates symptoms of ED [38]. During aging, a notable decrease in SMCs and an obvious increase in collagen accumulation were observed [5]. Therefore, augmenting the amount of smooth muscle and attenuating collagen deposition is pivotal to ameliorate ED associated with aging. It has been reported that transplantation of MSCs upregulates smooth muscle and augments the erectile response [14]. MiR-145 has been demonstrated to be a critical regulator of smooth muscle growth, differentiation, and function [37, 39]. Consistently, we showed here that treatment with miR-145-overexpressing BMSCs significantly increased the amount of smooth muscle, as indicated by KLF4 staining and decreased collagen and MMP2 in aged rats.

To further understand the mechanism of miR-145-expressing BMSC-mediated amelioration of ED, we examined changes in TGF-β signaling. TGF-β signaling is a well-described smooth muscle differentiation activator that contributes to miR-145-induced smooth muscle cell differentiation and matrix synthesis blockage. Smad2 is a critical mediator of TGF-β signaling. Consistent with previous results, we found that the phosphorylation of Smad2 was significantly decreased after treatment with miR-145-overexpressing BMSCs, confirming the involvement of TGF-β signaling in the action of miR-145. We further demonstrated that TGFBR2 is a direct target of miR-145.

## Conclusion

Collectively, administration of BMSCs overexpressing miR-145 significantly augmented the erectile response in aged rats with ED. We further revealed that miR-145-overexpressing BMSCs exert their therapeutic effect via increasing SMCs, which was coordinated by TGF-β signaling mediating the downregulation of collagen 1 and MMP2 .

## Data Availability

For data availability, please contact the corresponding author.

## Abbreviations

PDE5: Phosphodiesterase type 5
MiR: MicroRNA
KLF4: Kruppel-like factor 4
BMSC: Bone marrow-derived stem cell
SMCs: Smooth muscle cells
TGFβ: Transforming growth factor β
ICP: Intra-cavernous pressure
MAP: Mean arterial pressure
MMP2: Matrix metallopeptidase 2

## References

[1] Sanchez-Cruz JJ, Cabrera-leon A, Martin-Morales A, Fernandez A, Burgos A, Rejas J (2003). Male erectile dysfunction and health-related quality of life. Eur Urol.

[2] Nehra A, Steers WD, Althof SE, Andersson KE, Burnett AL, Costabile RA, Goldstein I, Kloner RA, Lue TF, Morales A, Rosen RC, Shabsigh R, Siroky MB, King L (2003). Third international conference on the management of erectile dysfunction: linking pathophysiology and therapeutic response. J Urol.

[3] Nicolosi A, Glasser DB, Kim SC, Marumo K, Laumann EO (2005). Sexual behaviour and dysfunction and help-seeking patterns in adults aged 40-80 years in the urban population of Asian countries. BJU Int.

[4] Haczynski J, Lew-Starowicz Z, Darewicz B, Krajka K, Piotrowicz R, Ciesielska B (2006). The prevalence of erectile dysfunction in men visiting outpatient clinics. Int J Impot Res.

[5] Ferrer JE, Velez JD, Herrera AM (2010). Age-related morphological changes in smooth muscle and collagen content in human corpus cavernosum. J Sex Med.

[6] Sadovsky R, Miller T, Moskowitz M, Hackett G (2001). Three-year update of sildenafil citrate (Viagra) efficacy and safety. Int J Clin Pract.

[7] Lin G, Hayashi N, Carrion R, Chang LJ, Lue TF, Lin CS (2005). Improving erectile function by silencing phosphodiesterase-5. J Urol.

[8] Peixoto CA, Nunes AK, Garcia-Osta A (2015). Phosphodiesterase-5 inhibitors: action on the signaling pathways of neuroinflammation, neurodegeneration, and cognition. Mediat Inflamm.

[9] De Young LX, Domes T, Lim K, Carson J, Brock GB (2008). Endothelial rehabilitation: the impact of chronic PDE5 inhibitors on erectile function and protein alterations in cavernous tissue of diabetic rats. Eur Urol.

[10] Sahara M, Sata M, Morita T, Nakajima T, Hirata Y, Nagai R (2010). A phosphodiesterase-5 inhibitor vardenafil enhances angiogenesis through a protein kinase G-dependent hypoxia-inducible factor-1/vascular endothelial growth factor pathway. Arterioscler Thromb Vasc Biol.

[11] Loeb S, Folkvaljon Y, Lambe M, Robinson D, Garmo H, Ingvar C, Stattin P (2015). Use of phosphodiesterase type 5 inhibitors for erectile dysfunction and risk of malignant melanoma. JAMA..

[12] Yang J, Zhang Y, Zang G, Wang T, Yu Z, Wang S, Tang Z, Liu J (2018). Adipose-derived stem cells improve erectile function partially through the secretion of IGF-1, bFGF, and VEGF in aged rats. Andrology..

[13] Aziz MTA, El-Haggar S, Mostafa T, Atta H, Fouad H, Mahfouz S, Rashed L, Sabry D, Senbel A, Ali GA (2010). Effect of mesenchymal stem cell penile transplantation on erectile signaling of aged rats. Andrologia..

[14] Bivalacqua TJ, Deng W, Kendirci M, Usta MF, Robinson C, Taylor BK, Murthy SN, Champion HC, Hellstrom WJ, Kadowitz PJ (2007). Mesenchymal stem cells alone or ex vivogene modified with endothelial nitric oxide synthase reverse age-associated erectile dysfunction. Am J Physiol Heart Circ Physiol.

[15] Ying CC, Yang M, Zheng XM, Hu WL, Wang XH (2013). Effects of intracavernous injection of adipose-derived stem cells on cavernous nerve regeneration in a rat model. Cell Mol Neurobiol.

[16] Liu G, Sun X, Bian J, Wu R, Guan X, Ouyang B, Huang Y, Xiao H, Luo D, Atala A, Zhang Y, Deng C (2013). Correction of diabetic erectile dysfunction with adipose derived stem cells modified with the vascular endothelial growth factor gene in a rodent diabetic model. PLoS One.

[17] Milenkovic U, Albersen M, Castiglione F (2019). The mechanisms and potential of stem cell therapy for penile fibrosis. Nat Rev Urol.

[18] Fang JF, Jia CC, Zheng ZH, Ye XL, Wei B, Huang LJ, Wei HB (2016). Periprostatic implantation of neural differentiated mesenchymal stem cells restores cavernous nerve injury-mediated erectile dysfunction. Am J Transl Res.

[19] Cordes KR, Sheehy NT, White MP, Berry EC, Morton SU, Muth AN, Lee TH, Miano JM, Ivey KN, Srivastava D (2009). miR-145 and miR-143 regulate smooth muscle cell fate and plasticity. Nature..

[20] Xu N, Papagiannakopoulos T, Pan G, Thomson JA, Kosik KS (2009). MicroRNA-145 regulates OCT4, SOX2, and KLF4 and represses pluripotency in human embryonic stem cells. Cell..

[21] Elia L, Quintavalle M, Zhang J, Contu R, Cossu L, Latronico MV, Peterson KL, Indolfi C, Catalucci D, Chen J, Courtneidge SA, Condorelli G (2009). The knockout of miR-143 and -145 alters smooth muscle cell maintenance and vascular homeostasis in mice: correlates with human disease. Cell Death Differ.

[22] Albinsson S, Suarez Y, Skoura A, Offermanns S, Miano JM, Sessa WC (2010). MicroRNAs are necessary for vascular smooth muscle growth, differentiation, and function. Arterioscler Thromb Vasc Biol.

[23] Moreira ED, Lbo CFL, Diament A, Nicolosi A, Glasser DB (2003). Incidence of erectile dysfunction in men 40 to 69 years old: results from a population-based cohort study in Brazil. Urology..

[24] Albayrak Ö, Şener TE, Erşahin M, Özbaş-Turan S, Ekentok C, Tavukçu HH, Çevik Ö, Çetinel Ş, Ertaş B, Şener G. Mesenchymal stem cell therapy improves erectile dysfunction in experimental spinal cord injury. Int J Impot Res. 2019. 10.1038/s41443-019-0168-1 [Epub ahead of print]. 10.1038/s41443-019-0168-1 31273327

[25] Jeon Seung, Zhu Guan, Bae Woong, Choi Sae, Jeong Hyun, Cho Hyuk, Ha U-Syn, Hong Sung-Hoo, Lee Ji, Kwon Eun, Kim Hyo-Jin, Lee Soon, Kim Hey-Yon, Kim Sae (2018). Engineered Mesenchymal Stem Cells Expressing Stromal Cell-derived Factor-1 Improve Erectile Dysfunction in Streptozotocin-Induced Diabetic Rats. International Journal of Molecular Sciences.

[26] Sun X, Luo LH, Feng L, Li DS (2018). Down-regulation of lncRNA MEG3 promotes endothelial differentiation of bone marrow derived mesenchymal stem cells in repairing erectile dysfunction. Life Sci.

[27] Mangir N, Turkeri L (2017). Stem cell therapies in post-prostatectomy erectile dysfunction: a critical review. Can J Urol.

[28] Soebadi MA, Milenkovic U, Weyne E, Castiglione F, Albersen M (2017). Stem cells in male sexual dysfunction: are we getting somewhere?. Sex Med Rev.

[29] Yiou R, Hamidou L, Birebent B, Bitari D, Lecorvoisier P, Contremoulins I, Khodari M, Rodriguez AM, Augustin D, Roudot-Thoraval F, de la Taille A, Rouard H (2016). Safety of intracavernous bone marrow-mononuclear cells for postradical prostatectomy erectile dysfunction: an open dose-escalation pilot study. Eur Urol.

[30] Al Demour S, Jafar H, Adwan S, AlSharif A, Alhawari H, Alrabadi A, Zayed A, Jaradat A, Awidi A (2018). Safety and potential therapeutic effect of two intracavernous autologous bone marrow derived mesenchymal stem cells injections in diabetic patients with erectile dysfunction: an open label phase I clinical trial. Urol Int.

[31] Bai Y, Zhang L, Jiang Y, Ju J, Li G, Xu J, Jiang X, Zhang P, Lang L, Sadkovaya O, Glybochko PV, Zhang W, Yang B (2017). Identification and functional verification of MicroRNAs in the obese rat with erectile dysfunction. Sex Med.

[32] Li DS, Feng L, Luo LH, Duan ZF, Li XL, Yin CH, Sun X (2017). The effect of microRNA-328 antagomir on erectile dysfunction in streptozotocin-induced diabetic rats. Biomed Pharmacother.

[33] Wen Y, Liu G, Zhang Y, Li H (2019). MicroRNA-205 is associated with diabetes mellitus-induced erectile dysfunction via down-regulating the androgen receptor. J Cell Mol Med.

[34] Wang H, Ding XG, Yang JJ, Li SW, Zheng H, Gu CH, Jia ZK, Li L (2018). LncRNA MIAT facilitated BM-MSCs differentiation into endothelial cells and restored erectile dysfunction via targeting miR-200a in a rat model of erectile dysfunction. Eur J Cell Biol.

[35] Wang H, Xie Z, Hou T, Li Z, Huang K, Gong J, Zhou W, Tang K, Xu J, Dong S (2017). MiR-125b regulates the osteogenic differentiation of human mesenchymal stem cells by targeting BMPR1b. Cell Physiol Biochem.

[36] Huang J, Meng Y, Liu Y, Chen Y, Yang H, Chen D, Shi J, Guo Y (2016). MicroRNA-320a regulates the osteogenic differentiation of human bone marrow-derived mesenchymal stem cells by targeting HOXA10. Cell Physiol Biochem.

[37] Yeh YT, Wei J, Thorossian S, Nguyen K, Hoffman C, Del Álamo JC, Serrano R, Li YJ, Wang KC, Chien S (2019). MiR-145 mediates cell morphology-regulated mesenchymal stem cell differentiation to smooth muscle cells. Biomaterials..

[38] Rogers RS, Graziottin TM, Lin CS, Kan YW, Lue TF (2003). Intracavernosal vascular endothelial growth factor (VEGF) injection and adeno-associated virus-mediated VEGF gene therapy prevent and reverse venogenic erectile dysfunction in rats. Int J Impot Res.

[39] Albinsson S, Suarez Y, Skoura A, Offermanns S, Joseph M, Miano JM, Sessa WC (2010). miRNAs are necessary for vascular smooth muscle growth, differentiation and function. Arterioscler Thromb Vasc Biol.

